# Death from Abuse of Ardent Spirits

**Published:** 1844-10-05

**Authors:** 


### DEATH FROM ABUSE OF ARDENT SPIRITS.

Dr. Nicol details, in the *London and Edinburg Med. Journal*, the particulars of the *post-mortem* appearances presented by the body of a young man, who was found dead after drinking freely of whiskey. The principal morbid phenomena were found in the alimentary canal; on opening the stomach, an evident odour of ardent spirits was emitted. The whole of the mucous membrane, from about one half up the œsophagus, along the whole extent of the stomach, and for about eighteen inches down the intestines, was highly injected; the vascularity of the corrugated part of the stomach was of a deep crimson colour through its whole extent. The œsophagus and the intestines presented a similar appearance, but the colour was less intense, gradually fading until it merged into the normal straw-colour of the healthy structure. The uncorrugated plane, immediately preceding or adjoining the pyloric orfice was highly vascular, of a bright scarlet or vermillion colour, evincing arterial injection, while the rest seemed decidedly venous. There were several large patches of viscid mucus adherent to the more depending parts of the stomach, which were first supposed to be coagulable lymph, but there was not any erosion, extravasation, or sanguineous stain on any part of the exposed surfaces. The brain was apoplectic. The fluids contained in the alimentary canal were analysed, but not a trace of poisoning could be discovered.

